# The Peculiar People

**Published:** 1898-12-17

**Authors:** 


### THE PECULIAR PEOPLE.

The decision of the Court of Crown Cases Reserved, confirming the conviction in the recent case of the Queen v. Senior, raises questions which are not without interest as to what is legally meant by the term "medical aid." In giving judgment, the Lord Chief Justice is reported to have said that the meaning of the words "wilfully neglect" was very clear. "Wilfully" meant what was done "deliberately and intentionally," and it was clear that "neglect" meant "the absence of such reasonable care as an ordinary parent would reasonably use for the care and protection of his child." The learned counsel's proposition was that because this particular parent was in other respects an affectionate father, except with regard to this one thing, which was a necessity to the child in the condition he was in, he was to be held guiltless because of his peculiar views. Where was the line to be drawn if that was to be accepted? There was, in his lordship's judgment, ample evidence to convict the prisoner in this case, and he considered that the summing up of his learned brother was perfectly right. The other judges concurred.

Now this raises a question. It having been decided that medical aid must be provided, does that mean the only medical aid recognised by law—that is, the aid of someone registered according to law—or is it to be left open to all and sundry to perform some hocus-pocus with some inert substance, and thus protect themselves by a pretence at "medical aid," while they go on with their laying-on of hands and their Christian scientism and all the rest of it just as before?

We clearly have not yet got to the end of this matter. No one pretends that everybody is bound to have a doctor when ill. Every adult person can do exactly as he or she likes in the matter, and all that the law need concern itself about is to get some fairly accurate information as to the cause of death if the patient's experiment should end fatally. But with children the matter is different. For every child in this country some one or another is responsible, and if they do not provide it with necessaries they are guilty of neglect, and must be punished. Among necessaries, when a child is ill, is "medical aid," and the law having said this, we submit that the only medical aid the provision of which will protect people from a charge of neglect, is that given by those who have undergone the training and submitted to the examination appointed in accordance with the law, namely, registered medical practitioners. This ought not to require saying; but, in fact, it is the very next question.

